# Rapid Detection of SNP (c.309T>G) in the *MDM2* Gene by the Duplex SmartAmp Method

**DOI:** 10.1371/journal.pone.0060151

**Published:** 2013-04-02

**Authors:** Yasuaki Enokida, Kimihiro Shimizu, Jun Atsumi, Alexander Lezhava, Yuki Tanaka, Yasumasa Kimura, Takahiro Soma, Takeshi Hanami, Yuki Kawai, Kengo Usui, Yasuko Okano, Seiichi Kakegawa, Hiroomi Ogawa, Yohei Miyamae, Yohei Miyagi, Haruhiko Nakayama, Toshihisa Ishikawa, Yoshihide Hayashizaki, Izumi Takeyoshi

**Affiliations:** 1 Division of Thoracic and Visceral Organ Surgery, Gunma University Graduate School of Medicine, Maebashi, Gunma, Japan; 2 RIKEN Omics Science Center, Tsurumi-ku, Yokohama, Kanagawa, Japan; 3 Department of Clinical Oncology, Yokohama City University Graduate School of Medicine, Kanazawa-ku, Yokohama, Kanagawa, Japan; 4 Kanagawa Cancer Center Research Institute, Asahi-ku, Yokohama, Kanagawa, Japan; 5 Department of Thoracic Surgery, Kanagawa Cancer Center, Asahi-ku, Yokohama, Kanagawa, Japan; University of Nevada School of Medicine, United States of America

## Abstract

**Background:**

Genetic polymorphisms in the human *MDM2* gene are suggested to be a tumor susceptibility marker and a prognostic factor for cancer. It has been reported that a single nucleotide polymorphism (SNP) c.309T>G in the *MDM2* gene attenuates the tumor suppressor activity of p53 and accelerates tumor formation in humans.

**Methodology:**

In this study, to detect the SNP c.309T>G in the *MDM2* gene, we have developed a new SNP detection method, named “Duplex SmartAmp,” which enabled us to simultaneously detect both 309T and 309G alleles in one tube. To develop this new method, we introduced new primers *i.e.*, nBP and oBPs, as well as two different fluorescent dyes that separately detect those genetic polymorphisms.

**Results and Conclusions:**

By the Duplex SmartAmp method, the genetic polymorphisms of the *MDM2* gene were detected directly from a small amount of genomic DNA or blood samples. We used 96 genomic DNA and 24 blood samples to validate the Duplex SmartAmp by comparison with results of the conventional PCR-RFLP method; consequently, the Duplex SmartAmp results agreed totally with those of the PCR-RFLP method. Thus, the new SNP detection method is considered useful for detecting the SNP c.309T>G in the *MDM2* gene so as to judge cancer susceptibility against some cellular stress in the clinical setting, and also to handle a large number of samples and enable rapid clinical diagnosis.

## Introduction

The *p53* gene encodes a nuclear protein that plays a pivotal role of inducing growth arrest or apoptosis of cancer cells in response to cellular stress and such external stimuli as drugs and medical radiation exposure [1]–[3]. The function of *p53* is reportedly compromised in many human cancers [4]. MDM2 is known as an oncoprotein that binds to p53 protein and inactivates the tumor suppressor activity of *p53* [5]. It has been documented that a single-nucleotide polymorphism (SNP) in the *MDM2* promoter region, a T-to-G change at nucleotide c.309 (rs2279744) in the first intron, increases the binding affinity toward stimulatory protein 1 (Sp1) and results in higher expression levels of MDM2 protein [6]. In an *in vitro* study, it was reported that cells harboring homozygous alleles of 309G/G express higher levels of MDM2 protein, thereby reducing the tumor suppressor activity of p53. In humans, the SNP 309T>G in the *MDM2* gene was reported to be associated with an earlier onset of tumor formation in both hereditary and sporadic cancers [7]. Meta-analysis studies have revealed that the presence of SNP 309T>G in the *MDM2* gene is associated with the risk of cancer [8]–[11]. Case-controlled studies showed a potential association of this SNP with cancer susceptibility in response to cellular stress [12]–[14]. Moreover, it has recently been reported that this polymorphism in the *MDM2* gene is associated with the prognosis for several types of tumors, such as esophageal, pancreatic, and lung cancers [15]–[17].

In this study, to detect the SNP c.309T>G of the *MDM2* gene in clinical samples, we aimed to develop a new SNP detection method, named “Duplex SmartAmp.” The original SmartAmp method was developed as a rapid, simple, and cost-effective method [18]. Hitherto, this method was applied to SNP genotyping [19]–[22], detection of genetic mutations [23], [24], and rapid detection of influenza virus [25], as it provided a practical platform for genotyping and virus detection based on its unique isothermal DNA amplification reaction and simple procedure of sample pre-treatment. These advantages are owing to its asymmetric primer set and an enzyme, *Aac* polymerase [18], which has strand displacing activity and a high resistance to cellular contaminants.

More recently, we have developed exciton-controlled hybridization-sensitive fluorescent primers, named “Exciton Primers” or “Eprimers,” which function as sequence-specific dyes that significantly enhance the signal/noise ratio [26]–[28]. After hybridization to complementary sequences, the Eprimer provides a sequence-specific fluorescent signal for real-time monitoring of amplification reactions. Eprimers show high signal strength with low background leading to a superior specificity and sensitivity compared with the commonly used SYBR® Green I [28].

To further advance our technology, in the present study, we introduced novel primers as well as two Eprimers to detect both 309T and 309G alleles simultaneously in one tube. We examined the specificity and reliability of the new method by comparison with the conventional PCR-based method. We here present validation data obtained with the new method named “Duplex SmartAmp” and demonstrate its genotyping data with clinical samples of lung cancer patients.

## Results

### Development of Primers for the Duplex SmartAmp Method

**Figure 1:**
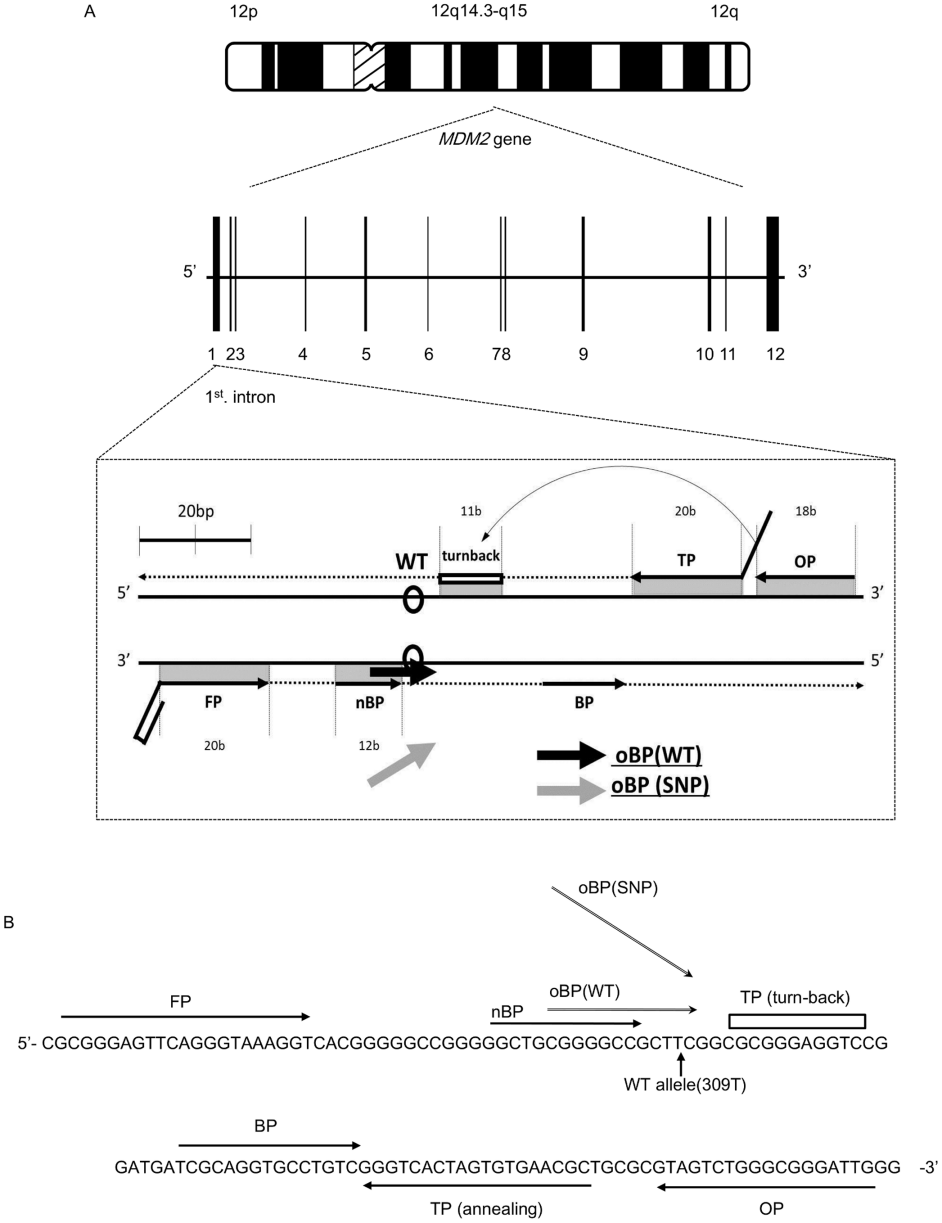
Strategy for Duplex SmartAmp-based detection of SNP (c.309T>G) in the *MDM2* gene. **A:** The *MDM2* gene is located on chromosome 12q14.3-q15, where SNP c.309T>G resides in intron 1. Annealing sites of the TP, FP, BP, OP, nBP, and oBP primers are shown in this schematic illustration. The size of each annealing site is numerically indicated in base (b) units. nBP, oBP(WT), and oBP(SNP) are designed to compete with each other to anneal with a site proximal to the SNP position. **B:** Horizontal arrows indicate annealing sites of primers. SNP (c.309T>G) is indicated by a vertical arrow.

Figure 1A depicts a schematic illustration of the strategy for detecting SNP (c.309G>T) in the *MDM2* gene by the Duplex SmartAmp method. To achieve high fidelity of SNP typing, we introduced two novel primers, namely, the outer Boost Primer (oBP) and the neutral Boost Primer (nBP), in addition to the standard SmartAmp primers (*i.e*., TP, FP, BP, and OP).

The reason for adopting such a SNP typing strategy is that GC-rich nucleotide sequences were found in both 5′- and 3′-regions adjacent to the SNP (c.309T>G), as shown in Figure 1B. In other words, the melting temperature of the GC-rich regions was so high that dissociation of the DNA strands became slower during the isothermal DNA amplification reaction. Two oBPs, *i.e*., oBP (WT) and oBP (SNP), were designed to discriminate T and G, respectively, at nucleotide c.309 between the wild type (WT) and SNP alleles (Figure 1B). These two oBPs were labeled with differently colored fluorescence dyes, as described below. The nBP served as a pacemaker for the detection reaction, where it is annealed at a locus proximal to the SNP position (Figure 1B).

The Duplex SmartAmp method requires the use of seven different primers: TP, FP, BP, OP, nBP, oBP (WT), and oBP (SNP). These primer candidates were selected on the basis of algorithms for free energy, probability of base-pairing, and product size range [29]. After extensive screening with a variety of synthesized oligo-nucleotides as primer candidates, we have selected one optimal set to use in the Duplex SmartAmp method for detecting SNP 309T>G in the *MDM2* gene. Table 1 summarizes the sequences of those primers comprising the optimal set. The genomic sequence located between the annealing sites of TP and FP (Figure 1A) is the target region that is amplified by the isothermal DNA amplification reaction.

**Table 1:** Sequences of primers developed for the Duplex SmartAmp method.

Primer	5′-DNA sequence-3′
TP	CGCGGGAGGTC AGCGTTCACACTAGTGACCC
FP	ACCTTCTATACCCTCAGAAGGT CGGGAGTTCAGGGTAAAGGT
BP	TCGCAGGTGCCTGTC
nBP	GGCTGCGGGGCC
OP	CAATCCCGCCCAGACTAC
oBP (WT)	5′- CGGGGCCGCZTC - 3′ (Z : Thymine labeled with thiazole pink)
oBP(SNP)	5′- CGGGGUCCGCTGC - 3′ (U : Thymine labeled with thiazole orange)

TP: The turn-back region is underlined.

FP: The folding region is underlined.

“Z” and “U” indicate thiazole pink- and thiazole orange-labeled thymines in oBP, respectively.

### Reaction of the Duplex SmartAmp Method

**Figure 2:**
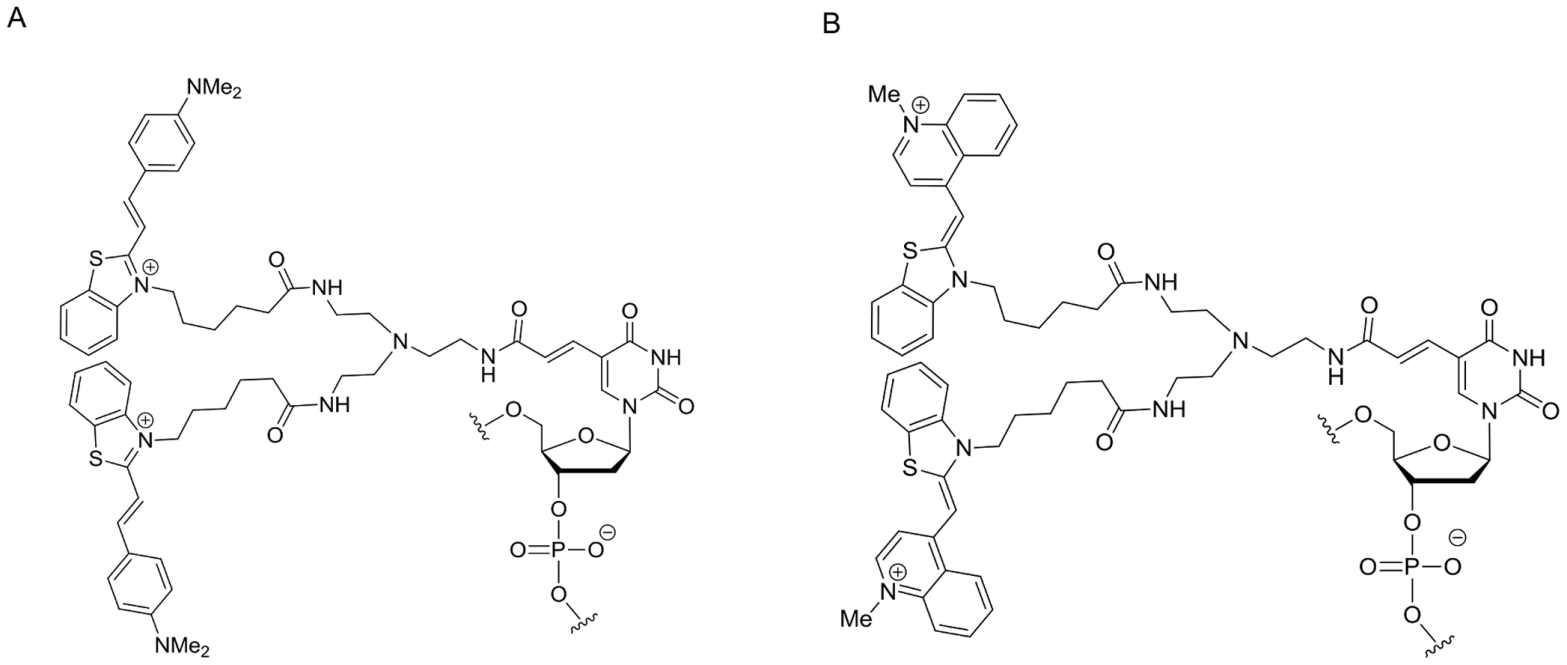
Chemical structure of thymine labeled with fluorescent dyes. A : Thiazole pink-labeled thymine. B : Thiazole orange-labeled thymine.

**Figure 3:**
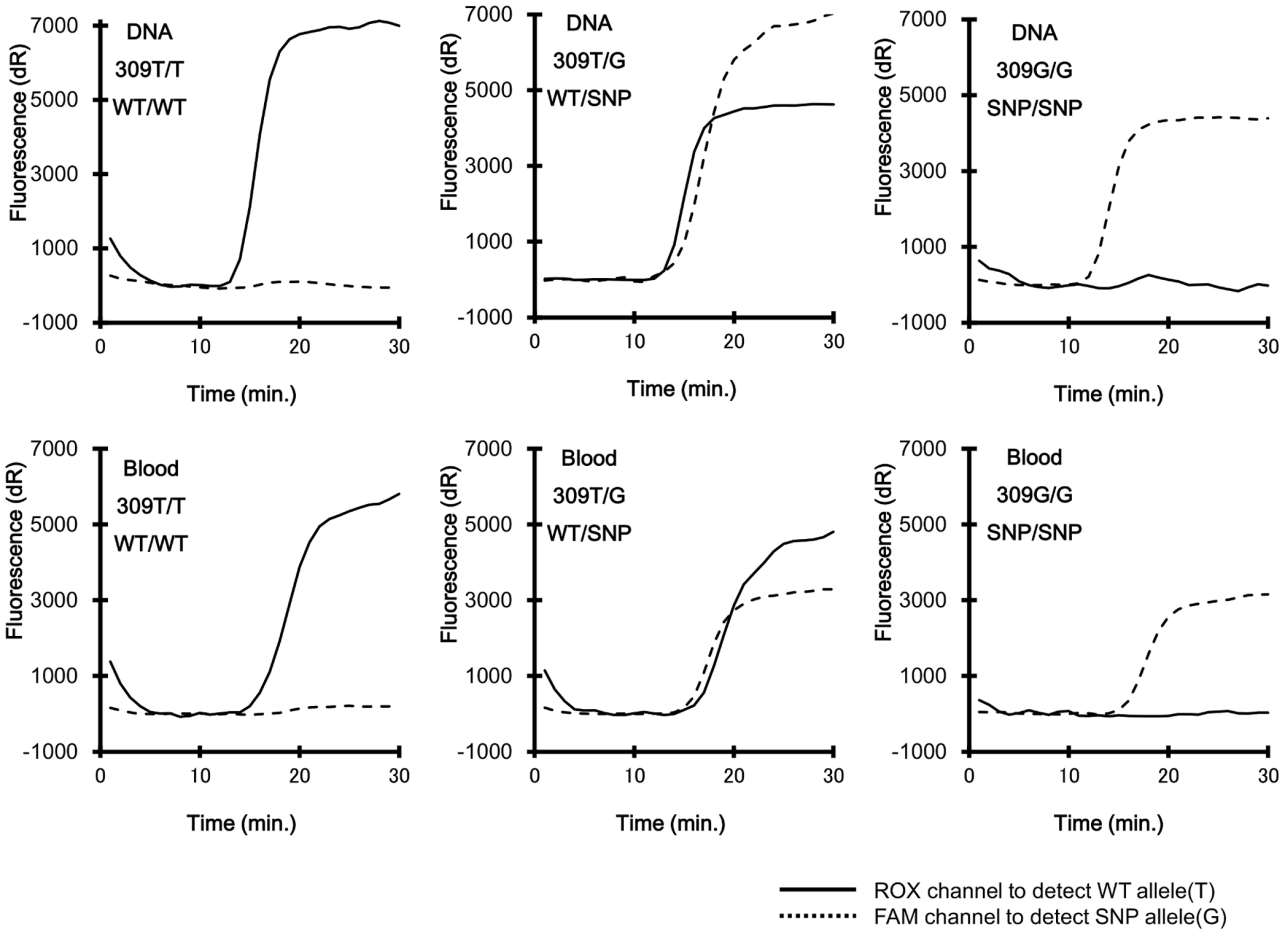
Duplex SmartAmp-based detection of SNP (c.309T>G) in the *MDM2* gene. Time courses of the Duplex SmartAmp assay reactions with genomic DNA samples (upper panels) and blood samples (lower panels). The experimental procedures are described in Materials and Methods.

oBP (WT) and oBP (SNP) were separately labeled with fluorescence dyes named thiazole pink and thiazole orange, respectively. One thymine in each of those oBPs was chemically linked with either thiazole pink or thiazole orange molecules at the position “Z” or “U” in the primer sequence (Table 1). Thyamine bases, except for both 3′- and 5′-terminals, can be labeled with exciton dyes. The sequence of Eprimers should be computationally designed to avoid any possibility of inter-primer dimmer formation and self-folding within a primer. Figure 2 represents the chemical structures of the thiazole pink (A) and thiazole orange (B) molecules. To detect the fluorescence of those dyes intercalated into DNA double strands during the Duplex SmartAmp reaction, thiazole pink and thiazole orange were excited at 585 and 492 nm, respectively, and their fluorescence was detected through the respective ROX (610 nm) and FAM (516 nm) filters in a real-time PCR machine Mx3000P (Agilent Technologies, Santa Clara, CA, USA). These primers selectively recognized the target sequence of SNP c.309T>G of the *MDM2* gene to discriminate homozygous 309T/T, heterozygous 309T/G, and homozygous 309G/G. As shown in Figure 3, the fluorescence intensity reached a plateau in 30 minutes over time for both the genomic DNA (upper panels) and blood (lower panels) samples.

**Figure 4:**
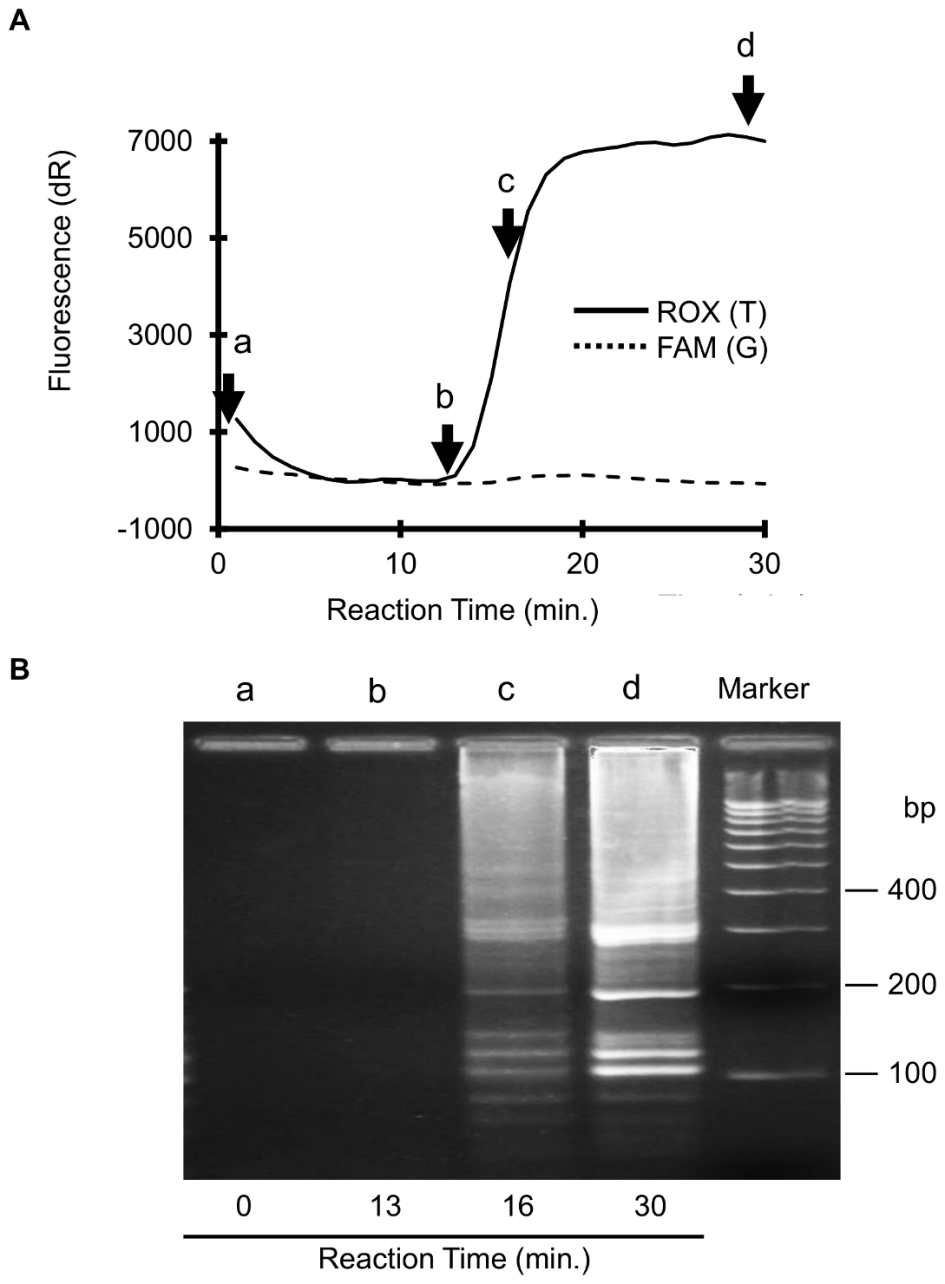
Analysis of DNA products formed in the Duplex SmartAmp reaction. A: Time course of typical Duplex SmartAmp reaction by real-time monitoring. B. Agarose gel electrophoresis of DNA products after Duplex SmartAmp reaction. We sampled aliquots of reaction mixtures at different reaction times (t = 0, 13, 16 and 30 minutes) and separated DNA products by agarose gel electrophoresis. The “Maker” represents a 100-bp DNA ladder.

To verify amplification products, by agarose gel electrophoresis, we analyzed the DNA amplicons formed during the reaction of Duplex SmartAmp assay. We sampled aliquots of reaction mixtures at different reaction times (t = 0, 13, 16, 30 min) and separated DNA products by agarose gel electrophoresis, as demonstrated in Figure 4. The multiple DNA bands pattern (t = 16 and 30 min) provides evidence that DNA products were formed through the self-primed amplification reaction as shown previously [18].

**Figure 5:**
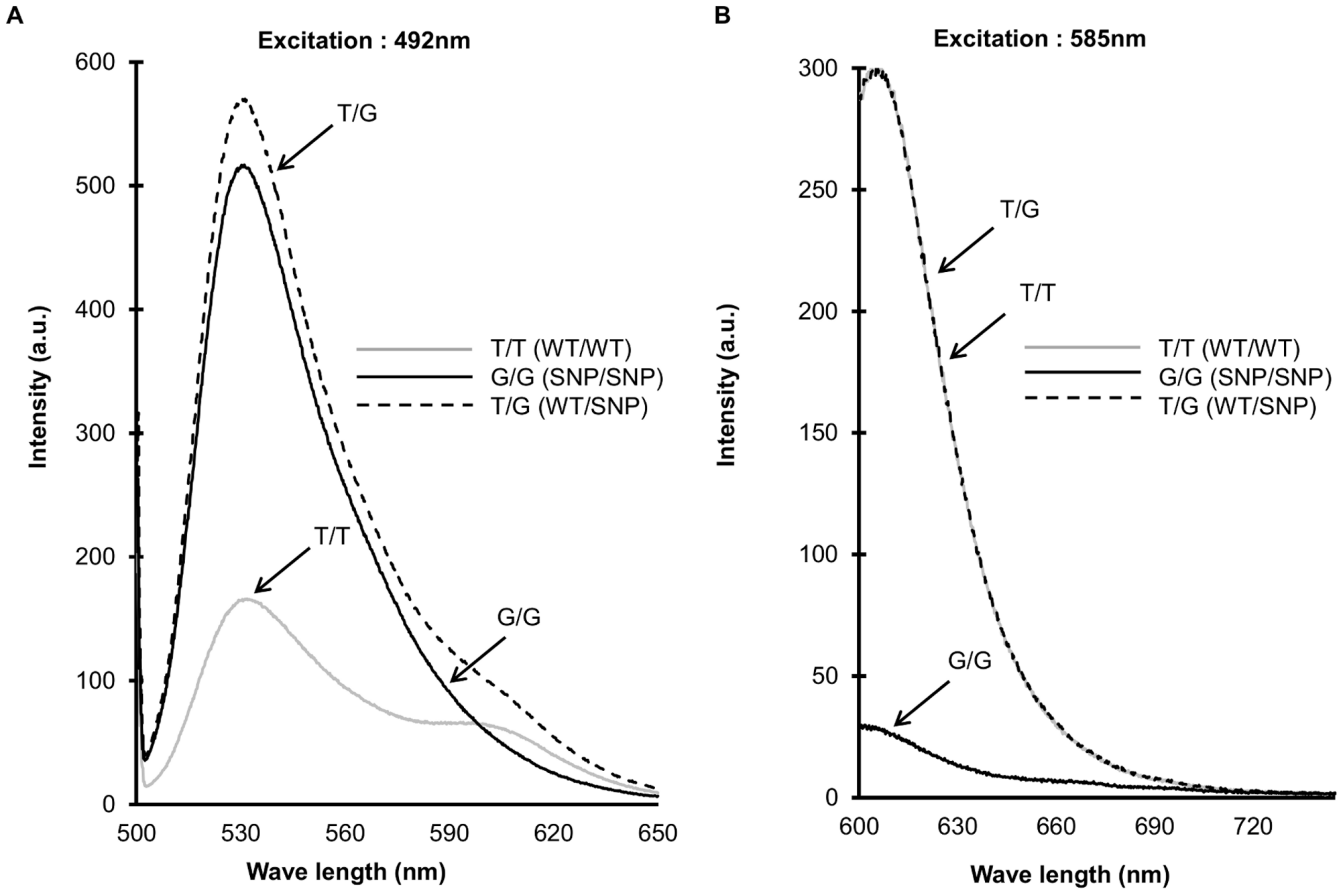
Fluorescence spectra of amplified products. The fluorescence spectra of amplified products were measured with excitation at 492 nm (A) and 585 nm (B) for oBP(SNP) and oBP(WT), respectively. The grey line, black solid line, and dotted line represent the fluorescence spectra of the amplification products of T/T (WT/WT), G/G (SNP/SNP), and T/G (WT/SNP), respectively.

To further examine those results obtained with the real-time PCR machine, the fluorescence spectra of the Duplex SmartAmp reaction products with genomic DNA were measured in an RF5300PC fluorescence spectrophotometer (Shimadzu, Kyoto, Japan) with excitation at 585 nm or 492 nm. As clearly shown in Figure 5, oBP (WT) linked with thiazole pink amplified the 309T allele, whereas oBP (SNP) linked with thiazole orange specifically amplified the 309G allele.

### Sensitivity of SNP Detection by the Duplex SmartAmp Method

In the clinical setting, a diagnostic tool for SNP detection from blood samples should have sufficiently high sensitivity, since the amounts of genomic DNA are often low in the blood samples from leukopenic patients, in particular, following chemotherapy. We examined the sensitivity of the Duplex SmartAmp method by conducting SNP typing with different genomic DNA concentrations (1.25, 2.5, 5.0, 10, 20, and 40 ng/µl) in the reaction mixture. As a result, we could detect each genotype (309T/T, 309T/G, and 309G/G) in the DNA concentration range of 2.5 to 40 ng/µL. No mismatch amplification was observed in those experiments (data not shown). The detection limit for genomic DNA is 2.5 ng/µL which is equivalent to 375 diploid cells/µL in the sample. Since the blood sample is diluted 3-fold at the preparation step and approximately 50% of genomic DNA is assumed to be extracted from leukocytes, the available DNA level corresponds to 2,250 cells/µL in the sample, being more enough for direct amplification.

### Comparison of the Duplex SmartAmp and PCR-RFLP Methods

**Figure 6:**
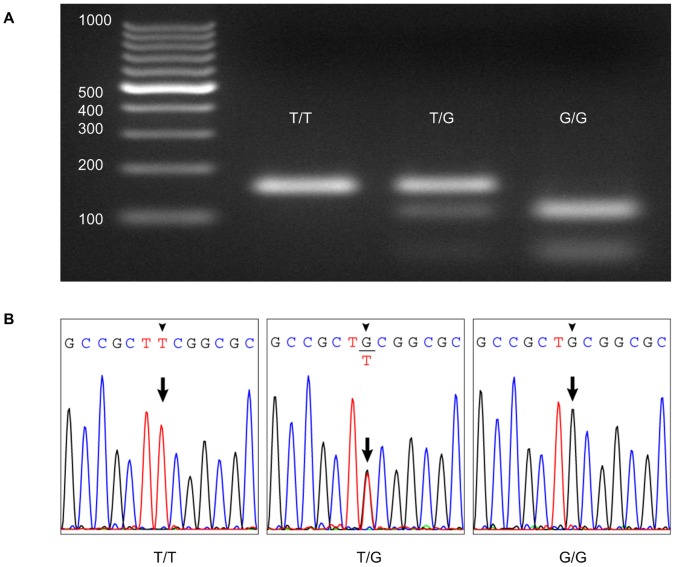
Comparison of results obtained by PCR-RFLP (A) and sequence analysis (B). Arrows indicate SNP positions.

From a total of 96 genomic DNA samples, we detected 309T and 309G alleles by both the Duplex SmartAmp method and the polymerase chain reaction-restriction fragment length polymorphism (PCR-RFLP) method (Figure 6). Furthermore, we used 24 blood samples and directly genotyped the SNP c.309T>G in the *MDM2* gene by the Duplex SmartAmp method. Genomic DNA was also prepared from the same blood samples and subjected to genotyping by the Duplex SmartAmp and PCR-RFLP methods. Table 2 summarizes the data from those experiments, which show that the results of the Duplex SmartAmp were 100% consistent with those of the PCR-RFLP method for both genomic DNA and blood samples.

**Table 2:** Comparison of results obtained by the Duplex SmartAmp and PCR-RFLP methods.

		Genomic DNA Samples : Duplex SmartAmp			Blood Samples : Duplex SmartAmp		
		T/T	T/G	G/G	T/T	T/G	G/G
**PCR-RFLP**	**T/T**	25	0	0	4	0	0
	**T/G**	0	46	0	0	15	0
	**G/G**	0	0	25	0	0	5

A total of 96 genomic DNA samples and 24 blood samples were subjected to the Duplex SmartAmp and PCR-RFLP assay methods to examine the genotypes of SNP (c.309T>G) in the *MDM2* gene.

### Clinical Validation of the Duplex SmartAmp Method

We further clinically validated the Duplex SmartAmp method by using 383 genomic DNA samples from patients with primary lung cancer (Table 3). The allele frequencies of T and G were 43.6% and 56.4%, respectively, which is consistent with a previous report on Japanese lung cancer patients [30]. As we used 96-well reaction plates, it took only one hour to both prepare samples and detect the 309T and 309G alleles for 48 samples by the Duplex SmartAmp method. In contrast, the PCR-RFLP method usually requires 80 minutes for the PCR, 90 minutes for the restriction enzyme reaction, and 30 minutes for electrophoresis [31]. Thus, as compared with this conventional method, the Duplex SmartAmp method markedly reduced the labor time and the handling steps for this large number of samples.

**Table 3:** Clinicopathological characterization of primary lung cancer patients as well as presence of SNP (c.309T>G) in the *MDM2* gene and allele frequency.

Characteristics		Patients (%)
Sex	Male	220 (57.4)
	Female	163 (42.6)
Age	Mean±SD	66.4±9.9 (years old)
Histological cell type	Adenocarcinoma	306 (79.9)
	Squamous cell carcinoma	40 (10.4)
	Others	37 (9.7)
SNP (c.309T>G) in the *MDM2* gene	T/T	82 (21.4)
	T/G	170 (44.4)
	G/G	131 (34.2)
Allele frequency	T	334 (43.6)
	G	432 (56.4)

## Discussion

### Duplex SmartAmp Method

The basic technologies of molecular diagnostics play a pivotal role in pharmacogenomics, particularly with respect to SNP genotyping. Diagnosis is integrated with therapy for selecting treatments as well for monitoring results. Cost-effective methods should be developed for genotyping, and it would be desirable to include this information in each patient’s medical chart as guidance for physicians to provide individualized treatment. The accurate measurement of allele frequency variations among population groups with different sensitivities to diseases and different responses to drugs is fundamental to genetic epidemiology. Errors in genotyping could markedly influence the clinical conclusions of pharmacotherapy. Thus, it is critically important to choose the appropriate method for accurate SNP detection.

To date, there are many different methods available for SNP typing, such as allele-specific hybridization (microarrays, AmpliChip CYP450), enzymatic cleavage (PCR-RFLP, invader assay), allele-specific PCR, mass-based detection (Sequenom), chemoluminescence (pyrosequencing), and fluorescence methods (direct sequencing and TaqMan) [32]. The central core components for all of these SNP typing methods rely primarily upon the stringency of DNA hybridization and the fidelity of enzyme reactions.

In this study, we have established the Duplex SmartAmp method to detect SNP c.309 T>G in the *MDM2* gene. The method has enabled us to detect this SNP in a single tube with a single drop (5 µL) of blood from a subject (Figure 3 lower panels). Furthermore, we have tested to use the buccal swab as well as nail plates and could detect genotypes by the Duplex SmartAmp method. The results were consistent with those detected with blood samples.

The distinguishing characteristics of this method are its simple procedure, isothermal reaction, low risk of contamination, high sensitivity and high specificity. In the Duplex SmartAmp method, we first introduced three primers, *i.e.*, nBP and two exciton dye-labeled oBPs. Together with the oBPs, nBP competitively hybridizes to the complementary sequence and provides the high fidelity of WT/SNP discrimination. Owing to these primers, *Taq* MutS [18] is no longer needed to suppress any background signals. We affirmed the WT- or SNP-specific DNA amplification results by PCR-RFLP (Figure 6), as well as by measuring the emission spectra of the reaction products (Figure 5). Thus, the Duplex SmartAmp method reduces by half the labor for development of primer sets, preparation time for assays, reagents, and template.

To realize the promise of individualized medicine, it is crucial to understand the molecular mechanisms underlying inter-individual differences in drug response and disease susceptibility. The Duplex SmartAmp has further advantages over the current real-time PCR system in its function to carry out genotyping by a simple system that is capable of handling a large number of samples. In the future, this achievement will allow medical staff to detect genotypes in the presence of patients easily, rapidly, and accurately, and hence to give feed-back for making a clinical decision based on individual patient genotypes.

### SNP c.309 T>G in the *MDM2* Gene

While the SNP (c.309 T>G) located in the *MDM2* gene was reported to contribute to individual genetic susceptibility to various cancers [8]–[17], at present there are conflicting results with respect to its association with cancer risk [33]–[35]. By the Duplex SmartAmp method, we analyzed a total of 382 samples from Japanese patients with primary lung cancer for the presence of a genetic polymorphism in the *MDM2* gene (Table 3). The frequency of the 309G allele was 56.4%, even higher than that of the 309T allele, which suggests that the SNP (c.309 T>G) is a relatively common polymorphism in the Japanese population. When adenocarcinoma and squamous cell carcinoma cases were compared, no significant difference was observed with respect to the ratios of T/T homozygote, T/G heterozygote, and G/G homozygote (Table 4). On the other hand, it is noteworthy that there is a large difference among African-American, Caucasians, and Asian populations with respect to the 309G allele frequency in lung cancer cases (Table 5). Asians, including Japanese, have higher frequencies of the 309G allele as compared with African-Americans and Caucasians. In this context, it would be of interest to analyze the ethnic group-dependent susceptibility to cancer.

**Table 4:** SNP (c.309 T>G) in the *MDM2* gene and allele frequencies in primary lung cancer patients.

	Number of patients (%)				Allele frequency (309G)
	Total	T/T	T/G	G/G	
Adenocarcinoma	306	67 (21.9)	137 (44.8)	102 (33.3)	55.7%
					
Squamous cell carcinoma	40	9 (22.5)	18 (45.0)	13 (32.5)	55.0%
					
Others	37	6 (16.2)	15 (44.4)	16 (42.1)	63.2%
					

**Table 5:** Ethnic differences of SNP (c.309 T>G) in the *MDM2* gene among lung cancer cases.

Ethnicity	Country	Sample size	T/T (%)	T/G (%)	G/G (%)	Allele frequency (309 G)	Reference
African-American	USA	133	110 (83.3)	20 (15.2)	2 (1.5)	9.1%	[36]
Caucasian	USA	1787	702 (39.3)	802 (44.9)	283 (15.8)	38.3%	[35]
Caucasian	USA	1026	419 (40.8)	472 (46.2)	135 (13.2)	36.2%	[34]
Caucasian	Norway	341	130 (38.1)	156 (45.7)	55 (45.7)	39.0%	[37]
Caucasian	USA	371	150 (40.4)	167 (45.0)	54 (14.6)	37.1%	[36]
Asian	China	717	166 (52.0)	373 (52.0)	178 (24.8)	50.8%	[36]
Asian	China	1106	249 (22.5)	561 (50.7)	296 (26.8)	52.1%	[38]
Asian	Korea	582	113 (19.4)	280 (48.1)	189 (32.5)	56.5%	[39]
Asian	Japan	377	68 (18.0)	183 (48.5)	126 (33.4)	57.7%	[30] *
Asian	Japan	383	82 (21.4)	170 (44.4)	131 (34.2)	56.4%	This study

* Squamous cell carcinoma.

### Concluding Remarks

In this study, we developed the Duplex SmartAmp for genotyping SNP c.309 T>G in the *MDM2* gene, and examined the reliability of this method in different ways. This method is considered useful for judging cancer susceptibility against some cellular stress in the clinical setting, and for handling many samples to explore a further clinical importance. The Duplex SmartAmp method can be more widely applied to the detection of other SNPs and is expected to provide a practical tool for point-of-care-testing. By using the Duplex SmartAmp method, the association of the SNP 309T>G in the *MDM2* gene with cancer risk will be studied in the future, and the corresponding results will be reported elsewhere.

## Materials and Methods

### Sample Collection

To carry out this clinical research, protocols for sample collection, sample anonymity, storage, and genomic DNA analysis were approved by the Institutional Review Board for clinical trials and the Ethical Committee for Human Genome Analysis at Gunma University, RIKEN Omics Science Center, and the Kanagawa Cancer Research & Information Association (KCRIA). Under written informed consent, blood samples were collected from patients with lung cancer who were treated surgically at the Gunma University Hospital (Gunma, Japan) and the Kanagawa Cancer Center (Kanagawa, Japan) during the period from January 2003 to August 2011. Overall, this clinical research was conducted according to the Declaration of Helsinki Principles.

### Sample Preparation

Peripheral venous blood samples from lung cancer patients were collected into tubes containing Na_2_EDTA. Genomic DNA was extracted with the use of the QIAamp blood kit (QIAGEN K.K., Tokyo) according to the manufacturer’s instructions. To perform the Duplex SmartAmp assay with blood samples, the peripheral venous blood samples were subjected immediately to the assay or stored at -80°C until used.

### The Duplex SmartAmp Assay to Detect SNP (c.309 T>G) in the *MDM2* Gene

A portion (∼5 µL) of the blood sample was mixed with two volumes (∼10 µL) of 50 mM NaOH solution and then heated at 98°C for 5 minutes to degrade RNA and denature proteins. On the other hand, isolated genomic DNA was diluted to a concentration of 20 ng/µL and then denatured at 98°C for 3 minutes. After chilling on ice, those samples (0.4 µL) were subjected to the Duplex SmartAmp assay, where the SNP-detection reaction was allowed to isothermally proceed at 60°C for 30 minutes in a Mx3000P PCR system (Agilent Technologies, Santa Clara, CA, USA). The reaction mixture (total volume of 10 µl for each) contained 3.2 µM FP and 3.2 µM TP, 1.6 µM BP, 1.6 µM nBP, 0.4 µM oBP(WT), 0.4 µM oBP(SNP), 0.4 µM OP, 1.4 mM dNTPs, 5% (v/v) DMSO, 20 mM Tris–HCl (pH 8.0), 30 mM potassium acetate, 10 mM (NH_4_)_2_SO_4_, 8 mM MgSO_4_, 0.1% Tween 20, and 4.8 units of *Aac* DNA polymerase (DNAFORM K.K., Yokohama, Japan). oBP (WT) and oBP (SNP) were Eprimers™ purchased from (DNAFORM K.K., Yokohama, Japan). During the Duplex SmartAmp assay reaction, thiazole pink and thiazole orange were excited at 585 nm and 492 nm, respectively, and their fluorescence was respectively detected through ROX (610 nm) and FAM (516 nm) filters.

### Data Analysis of the Duplex SmartAmp Assay

When the baseline-subtracted fluorescence intensity (Δ raw fluorescence: dR) was ≥1000 over the background level after a 30minutes-reaction, the amplification signal was judged to be positive. On the contrary, when the intensity was less than 1000, the amplification signal was judged as negative.

### Agarose Gel Electrophoresis to Analyze DNA Products after Duplex SmartAmp Reaction

The Duplex SmartAmp reaction was performed with genomic DNA (309T/T) as a template. Aliquots of the reaction mixture were taken out at different time points(0, 13, 16 and 30 minutes) and immediately heat-treated at 98°C for 5 minutes to terminate enzyme activity. Each sample was analyzed in 4.5% Nu Sieve CTG (Lonza, Basel, Switzerland) agarose gel and stained with ethidium bromide.

### Fluorescence Spectroscopy

After the Duplex SmartAmp assay, fluorescence spectra of the amplification products were measured with an RF5300PC fluorescence spectrophotometer (Shimadzu, Kyoto, Japan), where the sample was continually maintained at 60°C as for the Duplex SmartAmp reaction.

### PCR-RFLP and DNA Sequence Analysis

Genotyping of SNP (c.309 T>G) in the *MDM2* gene was carried out by PCR-RFLP as described previously [31]. The DNA sequence was analyzed with a laser-based automated DNA sequencer (ABI PRISM 3100 DNA Analyzer, Applied Biosystems Ltd., Tokyo, Japan).
